# Consistency and test–retest reliability of stepping tests designed to measure self-perceived and actual physical stepping ability in older adults

**DOI:** 10.1007/s40520-018-01112-3

**Published:** 2019-01-16

**Authors:** R. H. A. Weijer, M. J. M. Hoozemans, J. H. van Dieën, M. Pijnappels

**Affiliations:** Department of Human Movement Sciences, Vrije Universiteit Amsterdam, Research Institute Amsterdam Movement Sciences, Van der Boechorststraat 9, 1081 BT Amsterdam, The Netherlands

**Keywords:** Accidental falls, Self efficacy, Motor performance, Aged

## Abstract

**Background:**

Older adults with an incorrect perception of their physical abilities may fall more often, suggesting a need for tests to quantify self-perceived and actual abilities.

**Aims:**

To determine between-test consistency and test–retest reliability of three tests that measure self-perceived and actual stepping ability in older adults.

**Methods:**

Older adults performed three stepping tests, covering high (bar test) and far steps (river and step tests). We studied between-test consistency in the perceived ability and actual ability of 269 participants at each task and in the difference between these two (degree of misjudgment). We also studied test–retest reliability in 21 participants.

**Results:**

Perceived ability showed moderate consistency (*r* = 0.46–0.55, *p* < 0.001) and moderate-to-strong reliability [ICC(2,1) = 0.42–0.63, *p* < 0.03] for all tests. Actual ability showed strong consistency (*r* = 0.77, *p* < 0.001) and strong-to-excellent reliability [ICC(2,1) = 0.68–0.93, *p* < 0.001]. Degree of misjudgment was weakly consistent between two stepping far tests (*r* = 0.32, *p* < 0.001), but not consistent between stepping far and high tests (*r* = 0.05 and 0.06, *p* > 0.3). Test–retest reliability of the degree of misjudgment was poor-to-moderate [ICC(2,1) = 0.38 and 0.50, *p* < 0.05 on the two stepping far tests and ICC(2,1) = − 0.08, *p* = 0.63 on the stepping high test].

**Conclusions:**

Actual and perceived ability can be consistently and reliably measured across tests, whereas the degree of misjudgment is less reliable and consistent within individuals.

## Introduction

Thirty percent of older adults fall at least once a year and many of these falls lead to injury and fear of falling [1]. Delbaere and colleagues showed that when people have an incorrect perception of their own overall physical ability, this might increase the risk of falling [2]. Overestimation of one’s own abilities might result in taking too much risk in daily life and cause falls [3]. Underestimation might lead to physical inactivity, which, in turn, can amplify physical decline and fear of falling, increasing fall risk indirectly [4, 5].

Many falls happen during walking and the ability to make adequate steps to regain balance during walking is an important strategy for preventing such falls [6, 7]. When people have an incorrect perception of their own stepping ability, this may lead to more inadequate steps, which increases the risk of falling. For instance, when individuals perceive their maximum step length to be larger than their actual step length, they are overestimating their ability to deal with obstacles or to regain balance when challenged. To investigate whether an interplay between self-perceived and actual stepping ability is associated with and predictive of falls, we need easy to use tests to quantify an older individual’s self-perceived and actual ability, as well as a potential disparity between the two, the so-called misjudgment. These tests need to be reliable and show consistent results within subjects and between tests to be used to study or predict consequences and correlates of perceived ability, actual ability, and misjudgment [8].

Several tests have been described to determine one’s degree of misjudgment by measuring self-perceived and actual physical ability while walking or stepping [3, 8–11]. Studies using these tests showed that older adults tend to underestimate more frequently than they overestimate their actual abilities [9]. Furthermore, overestimation seems to be associated with falling [3, 9, 10]. However, consistency of the degree of misjudgment across tests has not yet been shown [8].

In the present study, we aimed to evaluate the between-test consistency and test–retest reliability of three stepping tests, designed to measure self-perceived and actual stepping ability, in older adults. These tests were either adapted from literature or developed for use in an ongoing prospective cohort study, Veilig in beweging blijven (VIBE) [8, 9].

## Methods

### Study design and study population

We analyzed data of 269 Dutch older adults who were enrolled in the prospective cohort study VIBE that started in 2017. The main aim of the VIBE study is to assess the modulating effect of self-perceived physical abilities on the relation between actual ability and prospective falls. Participants were community-dwelling older adults, who were recruited by flyers and newsletter adds in The Netherlands in 2017. They were included in the study if they were 65 years of age or older, if their Mini-Mental State Exam (MMSE) [12] score exceeded 19 out of 30 points and if they were able to walk at least 20 m (with walking aid if needed) without becoming short of breath or suffering chest pain. Participants were asked about their concern for falls using the Falls Efficacy Scale-International (FES-I) [13]. The FES-I is a self-report questionnaire measuring the concern for falling during everyday activities. It contains 16 items that can be answered on a scale from 1, not concerned, to 4, very concerned. The FES-I was filled out independently at home. Participants’ knee extension strength moment was measured, while participants sat on a chair. Their lower leg was strapped near their ankle to the hind legs of the chair, with a one-directional force transducer (KAP-E 2kN, A.S.T. GmbH Dresden, Germany) in the strap to measure knee extension strength, similarly as in the method described by Lord et al. [14]. The participants were instructed to extend their knee with maximum effort. We determined the maximum moment of three maximal knee extensions for left and right legs and summed them into a combined maximum knee extension moment. The lever arm used to determine the knee extension moment was determined as the distance between the joint line between the lateral epicondyle of the femur and the tibia plateau and the middle of the strap around the ankle at a location above the lateral malleolus.

Participants were also instructed to fill out the physical functioning (PF) subscale of the SF-36 Health Survey on both occasions, a questionnaire about self-reported health status. The PF subscale consists of ten items (e.g., ‘Does your health limit you in walking half a mile’) which can be answered with “Yes, limited a lot”, “Yes, limited a little”, or “No, not limited at all”. The total score of the PF subscale can range from 10 to 30 points, where 10 points indicate ‘limited a lot by health status’ and 30 indicate ‘not limited at all by health status’.

All participants performed the three stepping tests as described below at inclusion in the study. These data were used to test for consistency between tests. In addition, to determine the reliability of the stepping tests, 21 participants performed the tests again from 3 weeks to 3 months after the first test. The inclusion criteria of this subsample were stricter than those for the larger population, since they also participated in another study [15]. Participants were included if they scored more than 24 out of 30 points on the MMSE, had no self-reported cardiovascular, orthopedic, or neurological symptoms, and did not use any medication that hampered their stepping and walking abilities.

The raters, who assessed the participants’ self-perceived and actual stepping ability on the stepping tests, performed other physical and cognitive tasks with the participant immediately before the first rating and were not blinded to the first ratings for the reliability retest. The ethical committee of the Faculty of Behavioral and Movement Sciences of the Vrije Universiteit Amsterdam had approved the protocol (VCWE-2016-129) and all participants signed an informed consent form.

### Protocol

**Fig. 1:**
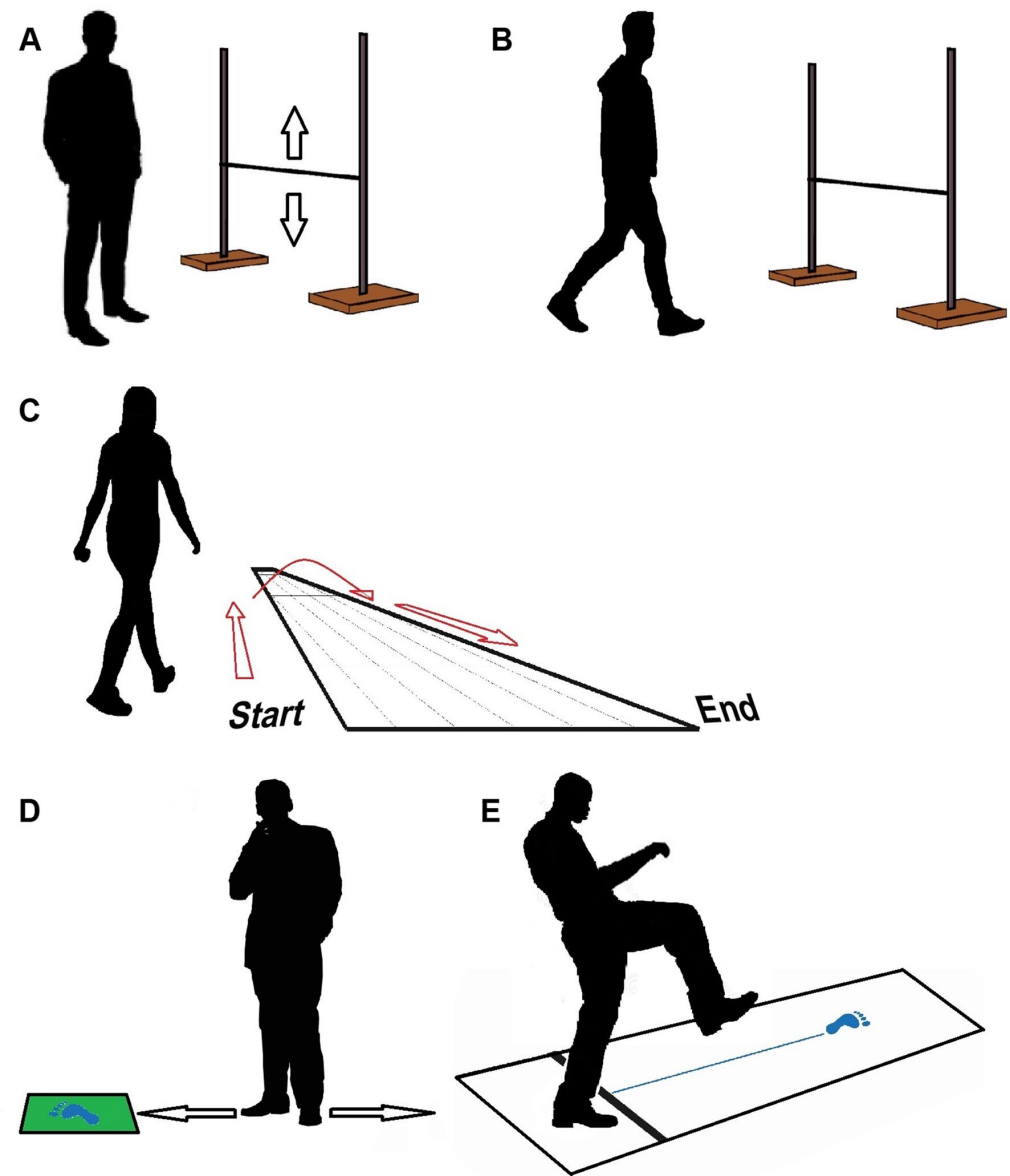
Test setup. **a** Bar test: perceived ability. **b** Bar test: actual ability. **c** River test: perceived ability. **d** Step test: perceived ability. **e** Step test and river test: actual ability

Participants performed three tests aimed at quantifying self-perceived stepping ability and actual stepping ability (Fig. 1): the bar test, the river test, and the step test, in that order. For the test–retest reliability, the first tests were assessed by one of three raters, who had had at least 1 day of training in assessing the tests. The reliability retests were assessed by a single rater with at least 3 months of training, who was also one of the three raters during the initial tests.

### Bar test

For the bar test, the participants were first asked to estimate the maximum height of a bar that they would be able to step over, keeping in mind that while their feet were on the floor, they had to be in a forward orientation (Fig. 1a). Hence, participants were only allowed to rotate their feet around the vertical axis when lifted off the floor. This restriction was used to ensure that all participants would step over the bar using the same strategy, namely by stepping over the bar in a forward instead of sideway manner. We asked for their perceived ability six times by moving the bar up and down and asking the participants, who stood at a 3 m distance from the bar, to say stop when they thought that the maximum height which they could step over was reached. Perceived ability was defined as the mean of the six chosen heights. Subsequently, we assessed their actual ability for this test, i.e., the maximal step height at which they could perform the test as instructed (Fig. 1b). We did this by letting them step over the bar repeatedly, which we put at an increasing height, starting at 10 cm with increments of 10 cm or 5 cm depending on the ease at which they cleared the bar. They were allowed two attempts at each height in case of a failure, before we lowered the bar by 5 cm. From this height, we again increased the height after each successful attempt, now with 2.5 cm increments. Actual ability for the bar test was thus defined as the maximum bar height that the participant could step over within two attempts while keeping the feet in a forward orientation as described above.

### River test

The river test was the second test that was performed. Participants had to cross a virtual river made out of a 12 m-long tapered sheet of paper, with a width of 1.84 m at one end and a width 0.31 m at the other end (Fig. 1c) [8]. They were instructed to start at the widest end of the river, cross the river, and return to the other side of the widest end of the river as quickly as possible without running or jumping and while making sure not to step onto the paper. We timed their performance, only to stimulate participants to act as quickly as possible. We defined their perceived ability for the river test as the width of the river at the location where they decided to step over it and their actual ability for the river test was determined as the actual ability in the step test (see below).

### Step test

For the step test, participants were asked to indicate the maximum length of a step which they thought that they could make onto a plastic non-slip mat placed on the floor (Fig. 1d). To do so, participants had to adjust their own position six times by either walking towards or away from the plastic mat which was placed at a semi-random distance by the rater, three times close to the participant and three times far away from the participant. We defined their perceived ability at the step test as the average of the six distances between their toes and the plastic mat. Subsequently, we measured their actual ability for the step test as their maximum step length by letting them take steps onto the mat, while placing the plastic mat increasingly further away (Fig. 1e). We started with a distance of 40 cm and increased the distance after a successful attempt with 5 cm or 10 cm depending on the ease with which the participant cleared the distance. After two failed attempts at a given distance, we decreased the distance by 5 cm. Next, participants were again asked to make an attempt, while we increased the distance by 2.5 cm. We defined the actual ability for the step test as the maximum distance which a participant could step within three subsequent attempts while being able to fluently continue the step with the trailing limb.

### Data analysis and statistical analysis

Statistical analyses were performed in R [R Core Team (2014), Vienna, Austria] and Matlab [MathWorks, Inc (2016), Nattick MA, USA]. Descriptive analyses were used to describe the demographic characteristics of the total sample and subsample of the study population. First, we tested the between-test consistency of perceived and actual stepping ability as well as the degree of misjudgment measured with and determined from the three stepping tests in the whole population. The degree of misjudgment was determined for each participant and each test by subtracting the actual stepping ability from the perceived stepping ability. Consistency was assessed using Pearson’s correlations within measures between tests. To interpret the correlation coefficients, we classified an *r* below 0.20 as very weak, 0.20–0.39 as weak, 0.40–0.59 as moderate, 0.60–0.79 as strong, and 0.80 and greater as very strong [16]. For each stepping test, we tested whether perceived and actual ability were linearly associated, as Kluft and colleagues [8] argued this to be a prerequisite for determining the degree of misjudgment through subtraction. We tested this by fitting multiple models to the data and checking whether the Akaike information criterion (AIC) for the linear model was lower than for the other models.

Next, we tested the test–retest reliability of the perceived ability, actual ability, and the degree of misjudgment for each of the three stepping tests on the subsample. Intra class correlation (ICC) estimates, standard error of measurement (SEM), and upper and lower limits of agreement (LoA) were calculated using R package ‘irr’ version 0.84 [17] based on a single-rating (*k* = 1), absolute-agreement, and a two-way mixed-effects model. We considered ICC estimates below 0.4 as poor, between 0.4 and 0.59 as moderate, between 0.6 and 0.79 as strong, and above 0.8 as excellent [18].

## Results

Table 1 shows the participant characteristics for the total group and the subset used for reliability testing.

**Table 1:** Participant’s characteristics

	VIBE cohort (*N* = 269)	Reliability sample (*N* = 21)
Age (years), median (IQR)	69.9 (67.7–74.8)	71.1 (68.8–75.8)
Female, *N* (%)	185 (68.8)	14 (66.7)
FES-I score, median (IQR)	19 (17–22)	18 (17–20)
SF-36 PF score, median (IQR)	29 (26–29)	29 (28–30)
Combined maximum knee moment (Nm)	82.4 (7.0)	85.1 (9.0)
MMSE score, median (IQR)	28 (28–29)	29 (28–30)
Perceived		
Bar (cm)	52.5 (13.5)	54.3 (12.0)
River (cm)	90.2 (18.8)	97.7 (11.2)
Step (cm)	77.8 (20.2)	80.4 (17.0)
Actual		
Bar (cm)	60.4 (10.8)	63.1 (9.2)
River and step (cm)	102.7 (19.9)	106.8 (15.8)

All values are means (standard deviation) unless otherwise noted. Combined maximum knee moments are the sum of the maximum knee moments for the left and the right leg

### Between-test consistency

**Fig. 2:**
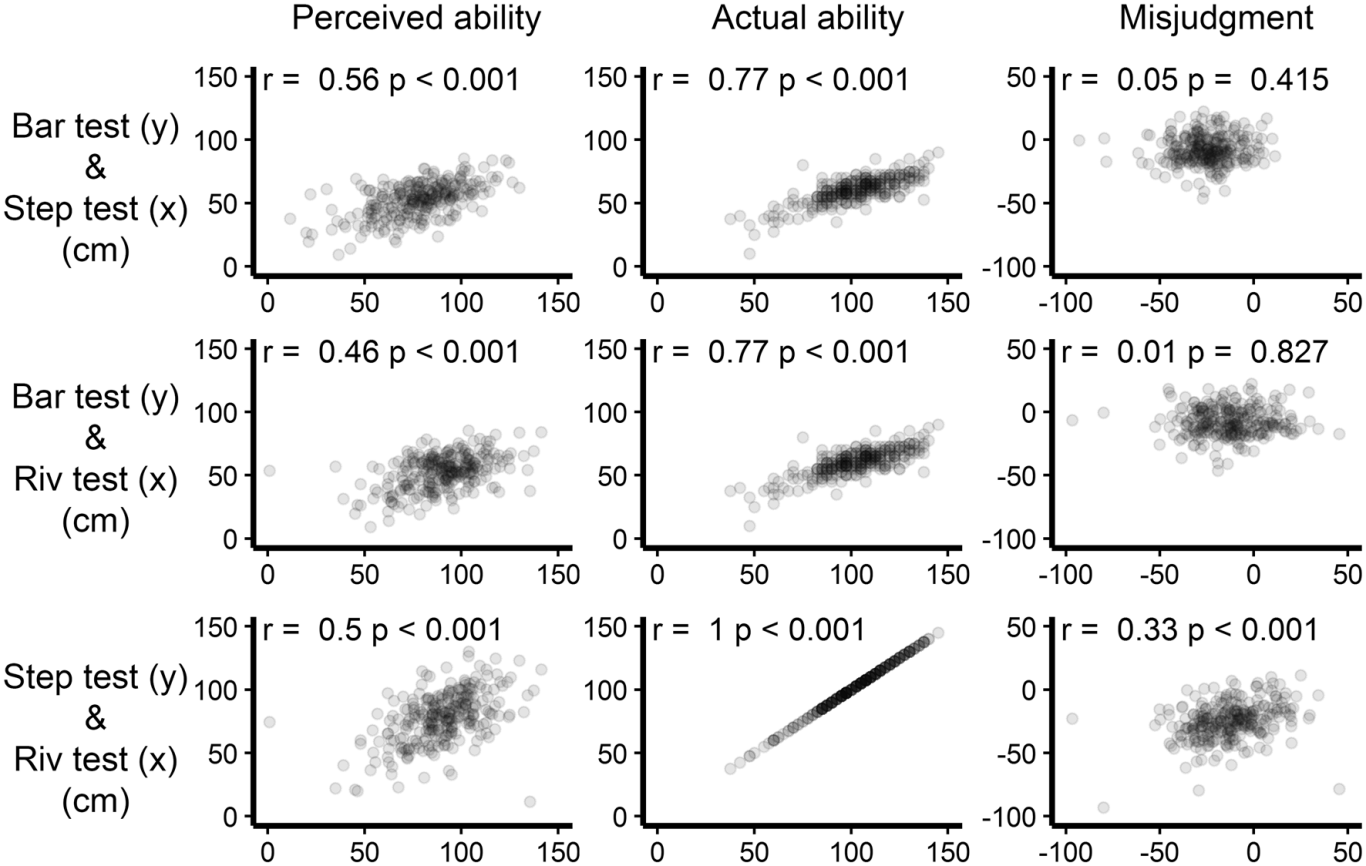
Consistency of measures across stepping tests. Axis labels are indicated on the left. ‘*y*’ indicates a vertical-axis label; ‘*x*’ indicates a horizontal-axis label. All axes represent centimeters. Negative degree of misjudgment indicates an underestimation, whereas positive values indicate an overestimation. Pearson’s *r* values and *p* values are presented in the top left corner of each graph

Correlations between the perceived abilities, actual abilities, and the degrees of misjudgment for all combinations of the three stepping tests are depicted in Fig. 2. Perceived ability measures showed moderate associations (*r*_perc: bar river_ = 0.46, *r*_perc: bar step_ = 0.56, *r*_perc: river step_ = 0.50, all *p* < 0.001), whereas, for actual ability, the scores on the stepping tests showed a strong association (*r*_act: bar step_ = *r*_act: bar river_ = 0.77, *p* < 0.001). As a requirement for determining the degree of misjudgment, we tested whether the relations between perceived and actual abilities were linear. The linear models (bar: AIC = 2056.5, river: AIC = 2254.6, step: AIC = 2176.2) performed better than the other models (next best models: bar: AIC = 2057.4, river: AIC = 2256.6, step: AIC = 2178.2) for all three stepping tests. This indicates that a linear relation between actual and perceived ability within the observed ranges is more likely than any other relation that we tested and that the degree of misjudgment can be determined by simple subtraction. The degrees of misjudgment for the river test and the step test were significantly, but poorly associated (*r* = 0.33, *p* < 0.001). For the degrees of misjudgment of the other stepping tests, only weak associations were observed.

### Test–retest reliability

**Fig. 3:**
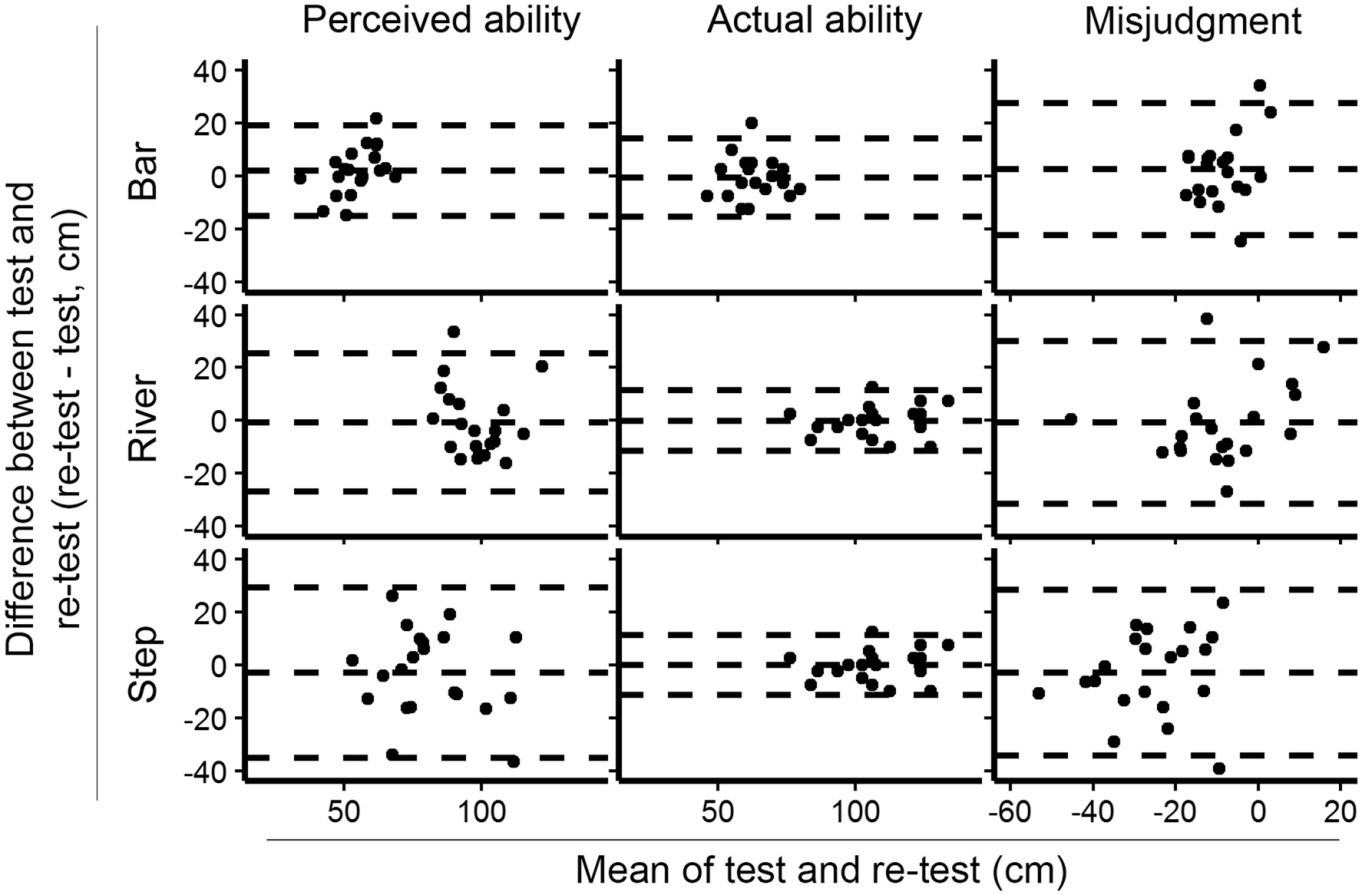
Bland–Altman plots. Columns represent perceived ability, actual ability, and the degree of misjudgment. Rows represent bar, river, and step tests. Dotted lines represent the upper and lower limits of agreement and the mean difference between the first rating and second rating

ICC(2,1) values, SEM, and upper and lower LoA are shown in Table 2, Bland–Altman plots can be found in Fig. 3. Only the actual ability for the step test showed excellent reliability [ICC(2,1) = 0.931, *p* < 0.001]. Actual ability on the bar test showed strong reliability [ICC(2,1) = 0.676, *p* < 0.001]. All perceived abilities showed moderate-to-strong reliability. The degree of misjudgment showed moderate reliability on the river test [ICC(2,1) = 0.501, *p* < 0.05] and poor reliability on the bar test [ICC(2,1) = − 0.075, *p* = 0.629] and the step test [ICC(2,1) = 0.377, *p* < 0.05].

**Table 2:** Reliability

*N* = 21	ICC(2,1) (*p* value)	SEM	Lower LoA	Upper LoA
Perceived ability				
Bar test, bar height (cm)	0.57 (0.002)	6.167	− 15.089	19.034
River test, river width (cm)	0.42 (0.029)	9.214	− 27.003	25.213
Step test, step length (cm)	0.625 (0.001)	11.515	− 35.172	29.087
Actual ability				
Bar test, bar height (cm)	0.676 (< 0.001)	5.247	− 15.45	14.26
River test and step test, step length (cm)	0.931 (< 0.001)	4.027	− 11.556	11.318
Degree of misjudgment				
p-a: Bar test	− 0.075 (0.629)	8.988	− 22.435	27.57
p-a: River test	0.501 (0.010)	10.88	− 31.64	30.087
p-a: Step test	0.377 (0.042)	11.202	− 34.195	28.347

ICC(2,1) estimates, SEM and limits of agreement for perceived ability, actual ability, and degree of misjudgment of each stepping test

## Discussion

We tested the between-test consistency and the test–retest reliability of three stepping tests designed to measure self-perceived and actual stepping ability in older adults. All tests showed consistency in participants’ perceived and actual abilities. Only the step and river test showed weak consistency in participants’ degrees of misjudgment. This was mainly because these measures of misjudgment were based on the same measure of actual ability of a far step, which will result in substantial covariance in the measure of misjudgment, as this is the difference between perceived and actual ability. Sufficient reliability was found to determine perceived and actual abilities, whereas the degree of misjudgment was moderately reliable for only one test and not reliable for the others.

### Consistency

The three stepping tests measured two forms of stepping ability that we expected to be highly related, stepping far and stepping high. Unsurprisingly, we found moderate and strong consistency of the actual and perceived stepping abilities, respectively, which supports the construct validity of the stepping tests. However, we found poor-to-no consistency for the degree of misjudgment determined from the differences between the actual and perceived abilities, similarly as shown for other comparable tests [8]. This indicates that older adults may overestimate or underestimate their physical abilities more or in a different direction for some tasks as they do for other tasks. Although our study was not designed to determine why this differs per task, we speculate that the perceived riskfulness of the stepping tests may have influenced the consistency of the degree of misjudgment over our stepping tests. During the bar test, participants had to balance on one limb while trying to lift their other limb over the bar, which may be perceived as more balance threatening than stepping far during the step test. The perceived riskfulness of the bar test when stepping over the bar may have limited them in performing to their actual maximum ability or altered their perception of their own abilities relative to the task at hand [19, 20]. The previous studies also showed a similar effect, where participants would change their behavioral decisions based on the perceived riskfullness associated with a task [21, 22]. This perceived riskfulness of a test may have led to variability in degrees of misjudgment for the stepping tests. Furthermore, personal experience with similar tasks may give participants a better understanding of the task at hand and of their likelihood to succeed at it [23].

### Reliability

Test–retest reliability of the actual ability on the step test was excellent, in contrast to only moderate test–retest reliability of the actual ability on the bar test. This latter finding was especially surprising as we expected actual ability to remain stable during a time period of 3 weeks–3 months. First, this unexpected finding might be explained by the specific instructions of the task. Although the instruction was to step over the bar without rotating the foot or feet that carried the body weight along their vertical axis, participants may have used minor rotations in one of the two occasions. Second, it is possible that factors such as tiredness or dizziness may have had a larger effect on the bar test than on the other tests, since the bar test relied more upon balancing ability, whereas the river test and step test relied more on muscle strength and agility.

Test–retest reliability of the perceived abilities was moderate-to strong, whereas test–retest of the degree of misjudgment was moderate or worse. Participants may have remembered if they overestimated or underestimated their stepping ability during the first rating. Subsequently, they may have been more or less cautious when indicating their perceived ability during the second rating. Hence, this could have affected reliability of both the perceived ability and the degree of misjudgment.

Another reason for the lower test–retest reliability of the degree of misjudgment could be our method of determining misjudgment. We quantified the degree of misjudgment by subtraction [8]. It could be argued that the degree of misjudgment may be dependent on the level of actual ability. People with a better actual ability may have a better perception of their abilities, since they might perform actions similar to the stepping tests more often in daily life than people with poorer actual ability [24]. In that case, a relative value for the degree of misjudgment, for instance by dividing perceived ability by actual ability, might be more suitable. However, this assumption was not supported in a previous study on perceived and actual gait ability in older adults [11]. Moreover, quantifying the degree of misjudgment as a ratio of perceived and actual ability will increase the variance of the misjudgment measure leading to a lower reliability.

Finally, although the subsample, used to assess test–retest reliability, was subjected to more strict inclusion criteria than the total sample, we found no relevant differences between the two samples, suggesting that the results from the between-test consistency analyses and the test–retest reliability analyses can be extrapolated to similar samples of community-dwelling older adults.

### Limitations

Our raters were not blinded for the result of the first measurements for the test–retest reliability. This might have led to higher ICC estimates as raters could have remembered this result at the time of the second measurement. Furthermore, participants may have recollected their performance at the first measurement, which could have influenced their perceived ability during the second measurement. Of these two limitations, the latter seems to be the most probable to have potentially influenced our results and may have led to an overestimation of the reliability of perceived ability and the degree of misjudgment.

The time between the first assessment and the second assessment ranged from 3 weeks to 3 months. 3 weeks may have been relatively short and participants may have had some memory of their ratings and performance on the first assessment, which could have led to high reliability. In contrast, in 3 months, time events in daily life that would influence participants’ perceived or actual ability are more likely to have occurred, which would lead to low reliability. However, as it is unlikely that many people suffered such a life event within that period of time we expect that this could not have led to biased results. Furthermore, the actual ability on the step test, which may also change due to life events, remained constant. During the second assessment, the physical condition of the participants was not assessed with methods other than the three tests. However, from Table 1, it can be seen that there was no relevant difference in physical functioning scores based on the questionnaires between assessments, suggesting relatively stable (self-reported) physical conditions. Besides these limitations, it should be taken into account that the participants in this study were reasonably fit. Applying the described tests in more frail populations may result in less of a ceiling effect.

### Practical considerations

We designed and adapted the three stepping tests to be used for predicting falls in older adults in future research. The advantage that these stepping tests have over the existing test is that they measure actual and perceived ability in a way that overestimation and underestimation can be directly quantified as a degree of misjudgment. As mentioned before, individual experience with situations that we aimed to simulate with our stepping tests may influence consistency and reliability of the degree of misjudgment. An individual’s experience in dealing with stepping far or high may be proportional to how active a person is in daily life and, thus, how often a person encounters similar challenges. Therefore, it may be beneficial for future studies that develop fall prediction models and consider using a combination of measures of perceived and actual ability, to also include a measure of exposure, for instance through ambulation monitors [25]. Either way, future fall prediction models that include the measures of perceived and actual ability, using the described tests, should consider not using a hard term such as the degree of misjudgment, but rather include an interaction term between the perceived and actual ability terms.

## Conclusion

Actual and perceived stepping ability can be measured consistently over tests and reliably over time, whereas the degree of misjudgment, defined as the perceived ability minus the actual ability, cannot be determined consistently over tests and reliably over time.
